# Human resources for health interventions in high- and middle-income countries: findings of an evidence review

**DOI:** 10.1186/s12960-020-00484-w

**Published:** 2020-06-08

**Authors:** Sophie Witter, Mariam M. Hamza, Nahar Alazemi, Mohammed Alluhidan, Taghred Alghaith, Christopher H. Herbst

**Affiliations:** 1 Queen Margaret University, Edinburgh, United Kingdom; 2 World Bank, Washington D.C., United States of America; 3 Saudi Health Council, Riyadh, Saudi Arabia

**Keywords:** Human resources for health policies, Middle-income countries, High-income countries, Physicians, Nurses, Allied health professionals, Literature review

## Abstract

Many high- and middle-income countries face challenges in developing and maintaining a health workforce which can address changing population health needs. They have experimented with interventions which overlap with but have differences to those documented in low- and middle-income countries, where many of the recent literature reviews were undertaken. The aim of this paper is to fill that gap. It examines published and grey evidence on interventions to train, recruit, retain, distribute, and manage an effective health workforce, focusing on physicians, nurses, and allied health professionals in high- and middle-income countries. A search of databases, websites, and relevant references was carried out in March 2019. One hundred thirty-one reports or papers were selected for extraction, using a template which followed a health labor market structure. Many studies were cross-cutting; however, the largest number of country studies was focused on Canada, Australia, and the United States of America. The studies were relatively balanced across occupational groups. The largest number focused on availability, followed by performance and then distribution. Study numbers peaked in 2013–2016. A range of study types was included, with a high number of descriptive studies. Some topics were more deeply documented than others—there is, for example, a large number of studies on human resources for health (HRH) planning, educational interventions, and policies to reduce in-migration, but much less on topics such as HRH financing and task shifting. It is also evident that some policy actions may address more than one area of challenge, but equally that some policy actions may have conflicting results for different challenges. Although some of the interventions have been more used and documented in relation to specific cadres, many of the lessons appear to apply across them, with tailoring required to reflect individuals’ characteristics, such as age, location, and preferences. Useful lessons can be learned from these higher-income settings for low- and middle-income settings. Much of the literature is descriptive, rather than evaluative, reflecting the organic way in which many HRH reforms are introduced. A more rigorous approach to testing HRH interventions is recommended to improve the evidence in this area of health systems strengthening.

## Background

Very few reviews exist which examine the policies and interventions adopted in high- and middle-income countries to tackle challenges related to the availability, distribution, and performance of human resources for health (HRH) [1]. More commonly, literature and evidence reviews have focused on low- and lower middle-income countries [2–4]; however, the challenges in higher-income settings are likely to be different—including, for example, higher dependence on imported labor [5–8]. In this article, we aimed to inform policy-makers in higher-income settings by examining the measures being taken to improve health workforce availability, distribution, and performance in higher-income countries and evidence of their effectiveness. Our review addresses the following questions:

1. What interventions have been tried in higher-income countries to address health workforce challenges, and what are the patterns of focus (in relation to production, recruitment, retention, distribution, and performance)?
2. How well supported by evidence on effectiveness are these different interventions?
3. Do any clear patterns emerge on differential effectiveness by occupational group or context?

## Methods

A guide for the evidence review was developed in advance by the research lead, including categories for intervention types, following a health labor market logic [9]; however, these were added to as additional approaches were identified in the studies. Searching and extraction were done by one primary researcher, supported by the research lead where queries arose.

### Search process and selection

A search of databases, websites, and relevant references was carried out in March 2019. For the peer-reviewed literature, search terms included the following:

1. Human resources for health: doctors/physicians, nurses, and allied health professionals^1^
2. Production/training, recruitment, financing, deployment, retention/attrition, management, motivation, productivity, quality, performance, and policy^2^
3. Middle- and high-income countries (using the World Bank classification).

**Fig. 1:**
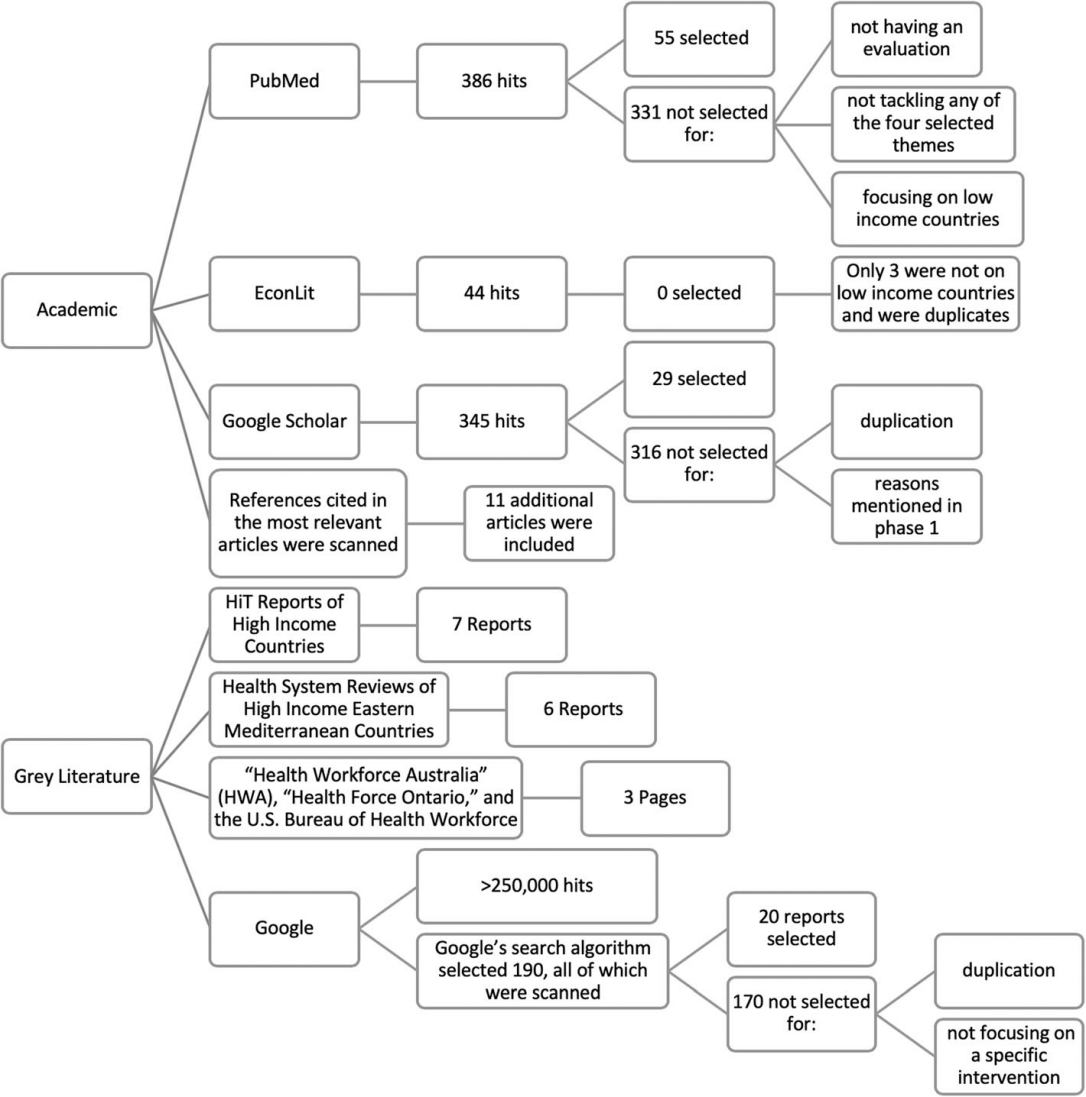
Flowchart of search methodology and selection. (Although some studies raise concern regarding the recall of Google Scholar, due to the limitation of not being able to view beyond 1000 search results [10], this was not of concern as search results were less than 1000. Google allows showing the “most relevant results,” which is based on an algorithm that omits duplicated and very similar results as well as accounting for history using the Google account)

Terms were searched in English, and dates were left open. In terms of methodologies, different study types were included as long as an element of evaluation of an HRH intervention was included. Figure 1 provides a flow chart of search methodology, number of hits, and the number of studies eventually selected for the review.

A final step involved scanning the references of papers that were very relevant. While most of the cited literature had already been reviewed, this yielded an additional 11 peer-reviewed papers.

For the grey literature search, a systematic approach was not feasible owing to the lack of comprehensive databases that include policy reports from all countries. All Health in Transition (HiT) reports for high-income countries were reviewed. As a second step, all Health System Reviews of high- and high-middle-income Arab countries were also reviewed, as there was a particular interest in learning about the Eastern Mediterranean region. This is a region with especially scarce compiled information, and it was a priority to compile all relevant information to aid policy-makers in HRH planning, which is picking up with the transformations occurring within the region [11]. From the Health System Reviews and HiT Reports, cited relevant reports were scanned to extract additional relevant information. Websites of organizations that are established to tackle the issue of HRH were also searched—these included “Health Workforce Australia” (HWA), “Health Force Ontario,” and the United States Bureau of Health Workforce.

Finally, the same search terms used for the peer-reviewed literature were used in Google. However, the number of hits was too many to go through, so further narrowing down was needed. Thus “OECD” was combined with “human resources for health,” and the relevant reports were obtained. This yielded a number of OECD working papers, which were all scanned, and the relevant ones included.

### Data extraction

All selected papers, both from the peer-reviewed and grey literature, were reviewed, and relevant information was extracted into a structured extraction sheet, which included the following: database, title, authors, year of publication, journal, country, study type, methodology details, context (rural/urban/underserved), occupational group addressed, definition of group addressed, description of challenges faced, intervention category and summary, impact category, impact summary, and conclusion/lessons learnt. There were also cells for implementation issues/challenges, and cost or cost-effectiveness; however, the majority of the studies did not address these issues.

### Study limitations

As the subject was broad, our focus was more on understanding the landscape than being exhaustive on any one topic. Our search terms may have missed some academic studies, and our review of the grey literature had to be limited. We also excluded most non-English language studies by use of English search terms.

## Results

### Bibliographic analysis

**Fig. 2:**
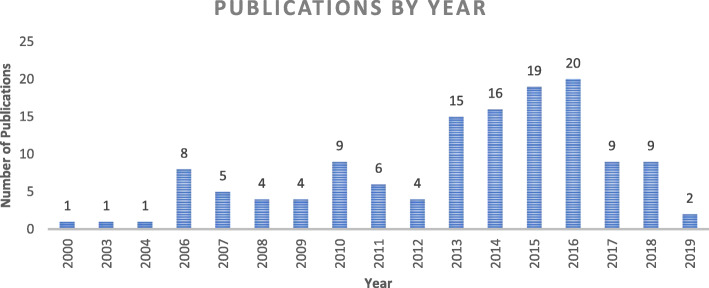
Publication by year

A total of 131 studies were included in the review. The analysis by year suggests a growth in the literature on this topic, peaking in 2016, which was (perhaps not coincidentally) the year in which the WHO global strategy on HRH: workforce 2030 was published [12] (Fig. 2).

These included studies on Australia, Bahrain, Belgium, Canada, China, Finland, France, Germany, India, Ireland, Italy, Japan, Jordan, KSA, Kuwait, the Netherlands, New Zealand, Norway, Oman, South Africa, Spain, Sweden, Switzerland, Thailand, Tunisia, United Arab Emirates (UAE), the United Kingdom of Great Britain and Northern Ireland, and the United States of America.

**Fig. 3:**
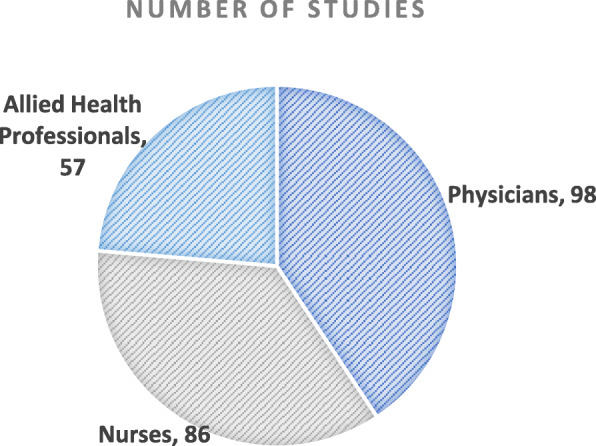
Number of studies by occupational group

The total aggregate number per occupational group is shown in Fig. 3.

The largest topic addressed was availability (84 studies), followed by performance (60 studies) and distribution (57 studies). Looking by intervention type (Table 1), within the production area, studies relating to training policies is the largest category identified (30 studies); on recruitment and retention, policies to reduce in-migration was the largest category (29 studies); in relation to distribution, educational incentives as a group had most studies (38 studies); on performance, training strategies is the largest topic (31 studies).

**Table 1:** Studies by intervention type

	Intervention	Number of studies that address it (broadly or specifically)	Summary of evidence
Availability (production + training)	1. Planning workforce and training needs	30	A variety of operational models developed and applied; complex data needs; limited evaluation of models identified.
	2. Training school capacity building	13	Important requirement; some documented examples, including for online learning.
	3. Attracting candidates	17	Wide variety of strategies, including outreach programs, selection policies, mentoring, and funding. For neglected specialties, more positive exposure during training may also be effective.
	4. Funding/financial access	10	Financing students can be effective, including for targeting underrepresented populations and directing them to less popular specialties and areas.
	5. Public/private and international partnerships for training	3	Detailed operational guidance has been developed as to how to implement international partnerships (mostly between low- and high-resource countries); no formal evaluations were identified however.
Availability (recruitment + retention)	1. Financing HRH	7	Limited evaluation studies but suggest that financing long-term positions can improve recruitment and retention.
	2. Targeted recruitment	14	To widen the pool, greater access to training, additional tax relief for continued work, phased retirement, flexible work schedules, and language support (for immigrant groups) can be effective.
	3. Improving HRIS	3	Important to support all HR functions; limited evaluations; implementation challenges noted.
	4. Policies to reduce outmigration	7	Provision of good working conditions, training opportunities, supervision, and manageable workloads are among the factors highlighted in some contexts.
	5. Increase in-migration of HRH	8	Bilateral partnerships and targeted visa programs are among the approaches shown to be effective, if this is the policy objective.
	6. Reduced in-migration to build domestic workforce	29	Increased training capacity and task shifting internally, and restrictive immigration and licensing rules for expatriates can be effective. To mitigate brain drain from low-income source countries, ethical codes have had at least some short-term effects.
	7. Increased retention	9	Preferences will be varied across cadres, age, location, and profile, so specific research is needed. For underserved specialties like primary care, it is important to provide good work/life balance and remuneration and build social status of role. Working hours and conditions, supervision, and access to training are typically important too. A balanced package should be provided. In some cases, task shifting to provide more support to clinical staff and delegate more routine tasks can support retention.
	8. Incentives to postpone retirement	5	Countries with aging populations and shortages have had some successes with incentives for health staff to postpone retirement, full-time or part-time, general or targeted to underserved areas.
Distribution	1. Educational interventions	38	There is a substantial evidence base that recruiting students from rural areas and exposing them to rural areas (positively) during training can increase later rural post-uptake and retention. More recently, “social accountability” medical schools have focused on reinforcing rural service ethic.
	2. Financial incentives	15	Financial support for those setting up and/or remaining in underserved areas may be effective, though evidence suggests that they need to be combined with support for living (e.g., housing) and working conditions.
	3. Non-financial incentives	15	A variety of strategies have yielded results in different contexts, including supporting CPD (including using remote learning for staff in remote posts), assisting spouses to find work, providing networks and personal support to reduce isolation, and mentoring.
	4. Bonding and contractual approaches	25	These appear to have had mixed success and/or are less studied. They include limited permits to serve by area to direct staff to shortage areas, bonding for a period as a condition for study grants, and contracts which allow for time away from the post (to rejoin families, where work stations are unattractive for them). Some countries also offer immigration opportunities for those willing to work in rural areas, though the longer-term effects on the local health labor market may be negative.
	5. Adjusting service provision model	10	Telemedicine and task shifting have been adopted in some locations to service rural, hard-to-staff areas. The former has not yet been extensively evaluated.
Performance	1. Training-related approaches	31	Pre-service training of course has a substantial impact on HR performance. A rich body of knowledge exists on good training practices, including the emphasis on problem-based learning, problem-solving, and interpersonal skills. CPD is receiving more focus for all health professionals and is often linked to relicensing or reaccreditation.
	2. Incentives and provider payment systems	10	Financial incentives are powerful, but complex. Most countries set wages centrally but recruit locally. Provider payment reforms are well documented—generally, mixed methods are recommended, with different approaches for primary and secondary care. Performance-based financing has been used in many countries, with some success and also challenges relating to cost-effectiveness and sustainability.
	3. Task shifting	9	Reasonably strong evidence that task shifting to nurses in advanced roles can provide good quality of care and outcomes, as well as playing a role in retention of physicians through reducing workload, though cost savings have typically been modest or non-existent. Resistance is high in some settings to changing professional roles.
	4. HR management	16	Decentralized HR management at the local level, along with effective deployment of HR management tools, is thought to improve performance, though evaluation evidence was lacking.
	5. Regulation of dual practice and absenteeism	8	Dual practice is common and can support public service (where pay is low and dual practice well-regulated) or disrupt it, also demotivation those who do not engage in it. Some of the common successful approaches include addressing the problem openly, revising incentives, improving working conditions, having professional value systems, and regulating work in the private sector. Failed interventions included prohibition and simply closing the salary gap. Absenteeism is seen as a warning sign to employers, who will need to understand the drivers. Changing organizational policies and culture, improving the workplace environment, and restricting private practice may be appropriate.

For the peer-reviewed literature, methodologies were mainly descriptive, with relatively few impact evaluations identified. Thirty-six studies used mainly descriptive statistics and regression analysis. Five studies used mixed method approaches, including interview analysis, focus groups, and descriptive analysis. Five used qualitative analysis of interviews. Finally, 22 concept notes and 24 systematic reviews were included.

### Overview of findings

The paper is organized according to four main themes, which were chosen as they reflect the main challenges faced by decision-makers in relation to HRH—(1) how to develop the supply through training, (2) how to engage and retain the right mix of health workforce, (3) achieving good distribution to match population needs, and (4) encouraging high-quality and responsive performance in their roles.

Table 1 shows the number of studies addressing each intervention category and provides a summary of the evidence.

### Production and training policies to tackle health workforce availability

Within the production and training area, studies were identified on the themes of workforce planning, development of training school capacity, policies designed to attract students from underrepresented areas, and to under-favored specialties, as well as training financing policies and training partnerships.

#### Planning workforce and training needs

Ensuring an adequate balance in the number and mix of health workers is essential for the proper functioning of health systems [13]. Several planning methodologies exist in order to minimize the possibility of an oversupply or undersupply [6, 14, 15]. This estimation however is becoming increasingly challenging due to the international mobility of the health workforce [16–18]. Evidence-based planning through modeling is used in many countries, including Australia, Belgium, Canada, Germany, Finland, Ireland, and Oman [6, 13, 14, 17, 19, 20].

The challenges identified were data related, as several data sources are needed and in many cases need to be collated [16] to make the projection models as accurate as possible. These models are constantly being revised and scenarios of planning changed [21–23]. With the diversity in approaches, different approaches to HRH planning will be appropriate for different jurisdictions depending on their respective contexts, availability of data, and the policy questions being faced [24].

One key long-term policy lever governments use to influence the supply of health workers is the regulation of admissions into education and training programs. Numerus clausus (i.e., closed number) policies [13] are usually implemented by fixing explicit quotas on admissions to medical and nursing education and post-graduate training programs nationally or regionally; some countries like the United States of America do not explicitly impose such quotas, but budgetary constraints at the national or sub-national level limit de facto the number of students admitted [13]. They are used in France [25], the Netherlands [26, 27], Australia [28], and Germany [29, 30]. Such policies tackle long-run supply, with lags in their effects, and need to be planned for using robust methodologies as explained in the projection methodologies section.

#### Training school capacity building

Increasing training numbers is theoretical and impractical without increasing school capacity, which is being done by many countries, even in less populated countries like Kuwait [31]. Capacity building in terms of faculty is more challenging; data shows that while there has been growth in health systems and policy research (HSPR) training over the past decade, only a minority of trainees move on to academic careers [32]. Another area discussed is online programs to increase capacity, which may be cost-effective; however, the challenge of high attrition arises due to the complexity of the learning tasks associated with eLearning and the lack of follow-up and interaction [33].

#### Attracting candidates

One challenge related to making more health workers available is the selectivity and targeting of medical schools, which makes the pool from which health workers emerge a lot smaller than it can be. This is a worldwide phenomenon being tackled by many countries with progress being made by the United Kingdom, Australia, and Japan through enforcing outreaching programs and providing funding [28, 34–36].

The trend of specialization in developed countries induces an undersupply of specific specializations, particularly general medicine, which is problematic due to the vast majority of patient interactions taking place at the primary care level [37] and that generalist intentions are often found to be a predictor of rural intentions/rural work [38].

One way to address this is to provide incentives (financial or non-financial) to encourage more physicians to choose to study general practice. This is being done in many OECD countries [13, 30, 39, 40]. Studies find that financial incentives are especially successful if the lack of choice of general medicine is a reflection of lower-income prospects. Financial incentives must be complimented by other reforms, especially curricular reforms [41].

Incorporating family medicine across all levels of education has also been shown to increase uptake of general and family medicine [39, 42]. It is important to understand medical students’ prioritizations when choosing residency programs as it varies by country; for example, in the UAE, future income was not found to be a significant contributing factor [43].

#### Funding/financial access

Public funding plays a key role, as in its absence, some training programs are forced to close, exacerbating issues of distribution and availability [44]. Government units with large funds to develop and implement policies tackling HRH training shortages exist in Australia [28]. Funds target specific trainings, including intern training, prevocational training, vocational training, specialist training, and inter-professional learning. Similarly in Japan, to address the undersupply of long-term care workers, publicly funded training is generally offered free-of-charge [45].

#### Public/private and international partnerships for training

Germany concluded a bilateral agreement with Vietnam in 2012, covering pilot projects for the training and recruitment of Vietnamese geriatric nurses, who would later serve in Germany. After 6 months of training, participants traveled to Germany to begin 2 years of professional training, accompanied by a program of integration and language courses [13]. International Medical Programs (IMPs), which are partnerships between training institutions in resource-limited and resource-rich areas to use education and training to build sustainable capacity, have a large literature. A comprehensive guide to designing successful IMPs for both parties to benefit has been compiled to aid in setting up this process [46]. One prominent successful example is the Makerere University, Uganda-Yale University Collaboration, a global health education capacity-building project designed to transfer critical training capacity [47].

### Increasing availability through recruitment and retention policies

Under recruitment and retention, we include discussion of a wide range of policies, including financing HRH, targeted recruitment, improving the HRIS, reducing out-migration, increasing in-migration, reducing reliance on foreign-trained health workers through training and immigration policies, and increasing retention through contractual policies, task shifting, and attractive packages for staff.

#### Financing HRH

Sometimes, the lack of funding and lack of full-time vacancies for graduates act as barriers to recruitment [48]. To overcome this, Canada implemented the New Graduate Nursing Initiative, which aims to promote the availability of permanent full-time positions for new nurse graduates and promote retention among Ontario’s Nurse Graduates to overcome the difficulty of transitioning professionally following training [49], particularly with the trend towards part-time and casual employment [48]. The Ministry of Health provides salaries for the first 6 months for newly recruited graduate nurses [50, 51]. The initiative has been evaluated through secondary data, focus groups, and interviews, showing success in stimulating new employment and retention of nurses. Nurses were pleased with immediate hiring and mentorship and decreased their considerations of emigration [49, 50, 52].

#### Targeted recruitment

The demand for long-term care (LTC) workers has been increasing, and recruiting LTC workers from underrepresented or inactive populations through greater access to training, additional tax relief for continued work, and phased retirement and flexible work schedules has been used. Some Finnish and Swedish LTC recruitment projects target male recruits. In the Netherlands, some recruitment strategies target ethnic minorities, secondary school students, or care workers that have left the profession. In Germany and Sweden, unemployed migrants are targeted with government-provided language courses [45].

#### Improving HRIS

Human resource information systems (HRIS) support a variety of human resource management (HRM) practices, including recruitment and performance management, and provide health leaders with crucial information guiding effective capacity planning and resource allocation. From evaluations, the most commonly realized benefits of HRIS are operational efficiency improvements. There are technological barriers though, specifically in relation to integrating with existing HR processes [53].

#### Policies to reduce outmigration

One issue contributing to the lack of available health workers is increased mobility, raising the importance of understanding and addressing push and pull factors, which are context-specific. In Ireland, “push” factors are poor working conditions, dissatisfaction with one’s work-life balance, and inadequate training and career opportunities, including low staffing levels leading to staff having to undertake non-core tasks [54, 55]. Post-graduate studies and continued professional development was one of the main reasons Irish workers chose to stay in Ireland [56]. Another finding was that greater length of staying abroad decreased the likelihood of returning to the home country [57].

#### Increase in-migration of HRH

Increasing immigration can take the form of formal partnerships, as practiced in Finland, through promoting work-based immigration of physicians and recruitment of foreign health and social care professionals; in Germany, recruiting Chinese caregivers [13]; and in Canada, recruiting internationally educated nurses and other migrant workers from the Philippines [58].

Increasing in-migration can also be done through sponsors and “green cards” as in the United States of America and Ireland [59]. An issue with increased in-migration is the “efficiency” challenge: how inflows need to be facilitated so that they are effective. Responses include integration programs, “fast tracking” work permit applications, multi-employer recruitment schemes to achieve economies of scale, approaches to place health workers quickly, and providing initial periods of supervised practice and language training, cultural orientation, and social support [17, 58].

One positive effect of increased in-migration is that it increases workforce diversity, and as patients in high-income countries are becoming more diverse, diversity, especially in nurses, is believed to benefit patients [60]. One issue that is very widely discussed is that increased in-migration results in an outflow from the sending country, which is usually a lower-income country, leading to the adoption of codes of ethics to ensure less harm occurs. In the United Kingdom, this resulted in considerable reductions in inflows of health professionals [61].

#### Reduced in-migration to build work domestic workforce

Increases in domestic training are the most widely cited approach to reduce reliance on foreign-trained health workers. The marked increase in domestic education and training efforts in many OECD countries over the past decade has already reduced considerably the inflows of foreign-trained physicians and nurses to these countries [13, 31, 59, 62–64]. Switzerland has focused on non-university-based health professionals [62]. One way to increase the domestic AHW workforce in particular is through training flexibility; allowing LTC workers to move easily between work and training in Denmark and the United Kingdom increased their availability [45].

Another approach is having more restrictive immigration policies. In France, legislative amendments have focused on the ability of physicians trained outside the European Economic Area to work in public hospitals [59]. Switzerland implements an immigration quota system for all foreign nationals [59]. In the UAE, licensing in particular is a major challenge as it takes 3 months minimum to have one’s qualifications recognized through a process that involves verification of qualifications [65]. Finally, while not directly tackling the issue of reliance on foreign workers, implementing an ethics code in the United Kingdom did reduce the inflow of migrant workers [61].

#### Increased retention

To minimize avoidable turnover, retention funding must be allocated under a framework that addresses *known* determinants of poor retention [66, 67]. This raises the importance of studying context-specific motivation factors.

Nursing was the most discussed in terms of turnover, which is why their retention is of particular interest. Some national strategies, like in Canada, are dedicated specifically for increasing interest in nursing careers [68]. To tackle shortages in general medicine, some countries, like Canada, France, and the United States of America, have recently increased post-graduate training places in general medicine to try to achieve a better balance between generalists and specialists. Other strategies focus on ensuring the attractiveness of the career financially and in working conditions. In the United Kingdom, three main factors that increased family medicine prestige were as follows: students being exposed early and often, family medicine being a fully recognized academic discipline, and graduates practicing in a health care system supportive of the discipline (e.g., good remuneration, easier work/life balance) [13, 37]. Finally, decentralized management, along with rewarding quality patient care, is also considered a retention strategy [69].

##### Guaranteed employment

Contract-based employment for nurses is a major demotivator in Chinese hospitals, increasing turnover. Contract-based nurses had a higher burnout, job dissatisfaction, and intent to leave; approximately 30% leave within a year of being hired [70, 71]

##### Official task enhancement

In Belgium, new regulations clarifying the status and roles of nurse aides have been enacted to improve their working conditions [72]. This also aims to decrease paperwork, improve patient care, and decrease stress for staff in caring for a diverse patient mix [69].

##### Attractive packages for health workers

In Belgium, Germany, and the Netherlands, working time-related factors significantly affect intention to stay across all countries; working part-time hours and overtime, and having a long commute time decreased the intention to stay with the same employer [67]. Similarly in India, the environment and benefits were more important than income in physician satisfaction [73]. In Denmark, to increase the supply of long-term workers, new trainees are employed as salaried LTC workers, which provides them with hands-on experience. Similar initiatives are implemented in the United States of America, the Netherlands, and Australia, such as employers paying training fees or providing paid leave [45].

For nursing, increasing the number of nursing staff to reduce workload; improving staff pay, education and continuing education, and housing subsidization; and improving status, organization, and quality of the work, the balance between professional and private life, and remuneration all affected retention [68, 69, 72, 74, 75]. In Canada, studies concluded that policy initiatives need to be tailored for different ages, profiles, and locations of nurses to re-attract former nurses and to retain current nurses [22].

##### Task shifting to reduce workload

Task shifting policy refers to delegating health care tasks to less specialized, lower-cost health workers. Transfer of skills was found to have improved the likelihood that personal support workers would stay in home care because it increased their job satisfaction [76]. A study from Scotland found that nurse practitioners employed to take over some of the tasks performed by junior physicians at night could reduce the intensity of the physicians’ work by almost 50% [17]. Task shifting is being done nationally in Australia through the Practice Nurse Incentive Program [28] and in UAE where, for example, pharmacists took on additional health screening and monitoring activities [77].

#### Incentives to postpone retirement

Policies to prolong the working lives of physicians, such as incentives for postponing retirement like pension reform, are used by many OECD countries, including the Czech Republic, France, Italy, and Portugal [45]. In France, retired physicians had been permitted to continue working in private practice, with earnings up to a fixed ceiling while still drawing their pensions. In 2009, the ceiling was removed, increasing the number of retirement-age physicians by 300% [25]. In Denmark, older general practitioners (GPs) can receive a bonus for postponing their retirement age. Postponing retirement is sometimes also used to tackle distribution; for example, in Northern Jutland, a remote underserved area in Denmark, GPs also receive a bonus between the age of 62 and 65 [78].

### Policies to improve distribution

Rural areas suffer from chronic shortages of health professionals. Systematic reviews have shown that strategies to tackle this issue can generally be grouped into educational, financial, and non-financial incentives, regulatory and supportive strategies [66, 79, 80]. As part of this search, reports were also identified on telemedicine, task shifting, and targeted immigration policies focused on filling distributional gaps.

#### Educational interventions

##### Pipeline approach: attracting rural candidates

A lot of studies have been done to understand what characteristics encourage different health workers to serve in rural or underserved areas. One proven approach to rural workforce shortages is the pipeline concept, focusing on undergraduates studying in rural schools [81]. This led to many countries investing in rural-focused medical education programs to increase the supply of rural physicians [21, 41, 82–84].

In Australia, graduates with 3 years of previous Rural Clinical School (RCS) training were more likely to indicate rural areas as their preferred current work location [85–87], similarly in the United States of America, in the University of Missouri School of Medicine [88], in Louisville [89], and in Illinois [90]. Globally, the pipeline approach is also used by New Zealand [91], Germany, [92], and Northern Norway [93]. Pipeline approaches have also been used for allied health professionals, including pharmacists [94].

##### Area-based study quotas

Area-based study quotas prioritize students from the same region having shortages, based on their increased likelihood of continuing to serve in that region. Fifty-two percent of Japanese physicians who had graduated from medical school remained in the prefectures in which they attended medical school after 10 years [95]. This incentivized “home quota” system led to a policy of having at least one medical school in each prefecture. This, however, was not successful in France [13, 96].

##### Social accountability of medical schools

“Social Accountability of Medical Schools” is a recent concept that encourages the production of competent professionals who are capable of demonstrating a positive effect upon the communities they serve [40, 97, 98]. Examples include the Hull York Medical School in the United Kingdom and the University of New Mexico School of Medicine, in which students are encouraged to join general medicine. Northern Ontario and University of Tromsø’s school of medicine in Northern Norway have also achieved some success by focusing on social accountability [13, 99–101].

#### Financial incentives

Financial incentives focused on those willing to work in underserved areas may incur “wastage” by providing incentives to those who would have practiced in underserved areas regardless of government intervention [78, 102]. Despite this, many high-income countries are implementing financial incentives to open practices and serve in rural areas (including Australia, Belgium, Canada, Finland, France, Germany, South Korea, New Zealand, Norway, Sweden, Switzerland, the United States of America, and the United Kingdom).

In Germany, financial incentives for GPs opening their practice in underserved areas for the first time are offered, but there is no evaluation as to whether it has helped recruitment [78]. In France, providing for practice start-up costs and paying physicians fixed salaries to cover liability risk are two approaches used to help recruitment in underserved areas [25]. In Denmark, limits have been established for nurses setting up practice in areas with high density, and financial and material incentives are offered to encourage new practices in underserved areas [25]. In Ontario, Canada, the Northern and Rural Recruitment and Retention Initiative offers grants for a practice opening in a rural area [13].

Several programs exist throughout Australia, Canada, Germany, and France to incentivize physicians, especially GPs, to serve in underserved, usually rural, areas. Different options exist, such as one-time grants [103], annual grants [78], scaled reimbursement schemes, and support to rural communities and their practitioners [21, 28].

Financial incentives can also target physicians after several years of practice and/or at the end of their career to improve their retention and to encourage them to postpone their retirement [78].

Financial incentives may not be successful, being insufficient to compensate for longer hours and generally more difficult working conditions [25, 78]. Good working conditions and affordable housing may be more effective [104].

#### Non-financial incentives

One of the main incentives used to recruit and retain health workers in remote areas is by offering continued professional development (CPD), sometimes virtually [21, 73, 105]. CPD is the second reason why recruits choose rural programs in Australia, after financial incentives [106]. It was also one of the enablers in Canada [107], as well as the main reason for nurses’ retention in rural Northeastern Ontario [108].

The aim of having support systems and group practices is to reduce burdens and isolation. It is one of the highest predictors of job satisfaction and retention in rural areas [105]. Professional support was also one of the main enablers of considering rural practice for dental students in Canada [107]. Furthermore, professional and personal support measures consistently top the surveys analyzing choices and preferences for work in these areas [102].

This is used in France (*Maisons de Santé Pluridisciplinaires*) [25], in Japan [78], in Scotland, [78], and in Australia [106]. Support includes psychosocial support, burden sharing, CPD opportunities, mentoring, and spousal employment assistance

#### Bonding and contractual approaches

Some countries require practice permits to be reimbursed, and there are regional quotas on permits issued in order to incentivize serving in underserved areas. This is practiced in Germany for ambulatory care physicians and in Denmark for GPs [78].

Bonding schemes are in essence offering a scholarship with a term-defined practice requirement to increase the number of physicians within certain regions. These are used in Japan [13, 78], Australia [21, 28, 41], and the United States of America, specifically targeting Hawaii [13, 78, 109]. The results are heterogeneous. In Australia, only 50 of 1200 graduates have started the return-of-service obligation period [21]. In the United States of America, participation was a significant predictor of practice location [110]. In Japan, almost 70% of the graduates of Jichi Medical School remained in their home prefectures for at least 6 years after their obligatory service [102].

Targeted immigration policies to attract foreign physicians to the country sometimes focus on specific underserved areas [13]. In the United States of America, H-1C non-immigrant visas were targeted specifically at foreign nurses for areas with shortages [59]. Similarly, in Australia, provider number restrictions direct international medical graduates (IMGs) to work in districts of workforce shortage, many of which are in rural areas, for a period of at least 10 years [21, 78]. In Canada, for IMGs, getting clinical training in most of the provinces comes with a return-of-service requirement in a designated underserved area [78]. In general, return-of-service requirements have been shown to be successful in the United States of America as physicians entering practice without J-1 visa waivers in rural communities had a significantly higher retention rate than their visa waiver colleagues [111]

While targeted immigration policies may be a relatively easy-to-implement short-term solution, there is no evidence that they improve distributional issues in the long term. These policies also reduce the incentive to expand educational capacity and improve working conditions [112].

The fly in, fly out (FIFO) model tries to make working in remote areas less challenging. Physicians spend a fixed number of workdays at their work location, geographically remote from their home and families, followed by a fixed number of days back in their home location, not working. The use of FIFO physicians has helped increase the number of rural GPs in regional Western Australia since 2008 [78].

#### Adjusting service provision model

##### Telemedicine

With technological advancements, adequate levels of access with fewer physicians may be provided through telemedicine [25]. In Australia, a support program exists to aid physicians in adopting telemedicine [28], while in Abu Dhabi, implementation of telemedicine is increasing [7], and in British Columbia, it is widely used, though challenges arise in rural areas with “low technological capacity” [78].

##### Task shifting in underserved areas to non-physicians

Several examples exist of task shifting in rural areas to lessen workload of existing staff, as well as reducing the need for more staff. In Germany, physicians can delegate home visits of older patients to non-physician practice assistants [78]. In France, since 2009, the role of pharmacists has been expanded as part of the Health Care Reform Law. In Canada, registered nurses (RNs) provide care to approximately 6.6 million Canadians living in rural areas [108].

### Performance-related policies

Performance of the health workforce can be addressed through interventions at each stage of the labor market, including training (pre-service and in-service), pay and incentives, human resource management policies and organization of work, and regulatory approaches.

#### Training-related approaches

##### Pre-service training

Better education leads to better performance; in China, a higher percentage of baccalaureate nurses was strongly related to better patient outcomes [74]. This area is still in need of further development in terms of empirically linking pre-service education with availability, retention, and performance of HWs [81]. In a systematic review on quality of education, it was shown that factors that contribute to quality pre-service education are as follows: competency-based curricula, match of training to health priorities, effective skills in clinical teaching, identification of student learning needs, assessment of student learning, prioritization and time management, infrastructure for computer-aided instruction, and sufficient clinical practice opportunities in high-quality clinical learning environments [81].

##### Curriculum reform

The most commonly cited reform involved increased clinical training and case training, using both simulated and actual patient encounters to ensure medical students gain confidence interacting with patients and transitioning between training and work, including in Denmark, the United Kingdom, the United States of America, and Kuwait [31, 45, 113]. In rural areas, a virtual flipped classroom approach is sometimes taken when clinical training is not feasible in the workplace [114]. This approach consists of blended learning or problem-based learning through an immersion via virtual reality clinical training or clinical training in the workplace, which is currently not available for many rural nursing distance education students due to the lack of commitment to investment in staff and in technology, which requires great funding.

Other reforms being called for include problem-based learning and team-based training of different health professionals [115] and the need for faculty to learn new content, skills, and technologies, with a focus on clinical training [116].

##### Continued professional development and mandatory re-licensing

CPD is an important policy lever to ensure that the skills of practicing physicians and nurses are kept up-to-date in a context of changing technologies and job requirements. From a systematic review of 33 articles, CPD, specifically master’s degrees, was associated with improved leadership skills, better job performance, and improved skills [117]. Similarly, Masters in Public Health (MPH) graduates from six counties contributed to improving graduates’ careers and to building leadership in public health. A caveat though is that graduates were less likely to practice medicine, affecting availability [118]. Most OECD countries and some non-OECD ones therefore have CPD and re-licensing policies despite some drawbacks like less graduates practicing in clinics and graduates finding some difficulty applying theory and expecting more guidance [117, 119–121].

#### Incentives and provider payment systems

Payment methods affect the performance of health workers and generate powerful incentives that have the potential to improve or reduce efficiency, equity, quality, and patient satisfaction. Interventions most cited include decentralization, linking pay to performance, balancing payment methods, separating health care purchasing and provision, and increasing providers’ accountability for resource use [17]. In a study on eight OECD countries, in the short run, centralization of wages helped preserve employment and service capacity during crises or recessions. In the long term though, a continuation of these centralized wage setting measures may run counter to structural reforms in the hospital sector that seek to provide greater autonomy to hospital management, which is linked with better performance and is a condition for the implementation of pay for performance (P4P) [122].

Many countries are implementing a variety of designs of P4P, including the United States of America, the United Kingdom, Canada, Australia, France, and Italy [123]. From 128 evaluations, 120 of which were from the United States of America and the United Kingdom, P4P was largely associated with positive effects on clinical effectiveness and access and equity of care; however, less clear evidence is shown regarding coordination and continuity of care, patient-centeredness, and cost-effectiveness [124].

#### Task shifting

Expanding the roles of non-physician providers to relieve pressures on physicians is one of the most widely used methods, given the increase in pressure with increased demand for health care. It has occurred in many countries, including Canada, Chile, Finland, Ireland, the Netherlands, New Zealand, Slovenia, Spain, Sweden, Switzerland, and the United States of America [13, 17, 28, 125, 126]. In some countries, junior physicians take on more tasks, and some tasks have even shifted to newly developed jobs, which require less training, such as phlebotomists to take routine blood samples [17].

Nurses in advanced roles in primary care were associated with higher patient satisfaction, reduction in hospital admission, reduction in mortality, and more return visits. The results for patient quality of life were inconclusive [17, 69, 127, 128]. In terms of cost-effectiveness, results are either cost-neutral or may slightly reduce the cost of health service (cost reductions being offset by higher consultation frequency and more return visits) [129].

The success of nurses in advanced roles is conditional on legislation and regulation to remove barriers to extensions in their scope of practice. Canada, the Netherlands, the United States of America, Australia, and UAE have taken legislative steps to strengthen nursing and midwifery advanced roles such as nurse practitioners (NPs) [13, 28, 130]

#### HR management

The most widely used approaches for performance management are as follows: job descriptions, work programs (whereby the hospital established what is expected by providing a weekly or monthly description of scheduled work undertaken), hospital by-laws, selection and appointment procedures, written contracts, internal appraisal systems, continuing professional development, external peer reviews, and external recertification [17]. As with many other areas, empirical evidence for the effectiveness of such approaches is lacking. For decades, partnership working has also been developed as the approach to employee relations through trade unions, managers, and government, and involving staff in key decisions [131].

Due to the rising demand for and growing complexity of health care and due to the increasing use of technologies, the skill set required by health workers has changed. Specifically, there is an increase in the need for “transversal skills”—interpersonal skills, such as communication, teamwork, and openness to continuous learning. Transversal skills are harder to measure, leading to the adoption of new approaches to measurement, like self-reporting tools for skills and skills use assessment [13, 17, 132]. Several efforts exist to try to standardize performance measurement. The International Council of Nurses (ICN) offers a set of guides on clinical interventions and international classification of nurses, but currently, there is no global framework for nurse competences or nurse skills [17], although one does exist for midwives [133].

#### Regulation of dual practice and absenteeism

The global issue with dual practice, particularly for nurses, is that with a secondary job, fewer hours are allocated to primary, public employment [134]. The main drivers for nurses to engage in dual practice were the need to increase earnings and for flexibility [135]. A caveat though is that by allowing public sector health workers to supplement their income, it may be easier for the public sector to keep their skilled health workers [134]. Interventions and regulations have an impact on the retention of those who do not engage in dual practice as it gives a sense of fairness [73] but must be context-specific and based on whether there is a salary gap that is exclusive to health sector [136].

Increasing job satisfaction and organizational commitment were good strategies for reducing absenteeism and turnover intentions of nurses [137]. The main findings of a systematic review [138], which included studies conducted in twelve high-income countries, is summarized in Table 2.

**Table 2:** Regulatory mechanisms against absenteeism [138]

Regulatory mechanism	Findings
Changes in organizational culture, including attendance policies	Effective and increased performance, reduced absenteeism
Restriction or prohibition of private practice	Workers only engaged in public sector work
Changes in employment contracts from fixed to permanent posts	Higher absence rates for those in permanent posts. Higher job security, but higher rates of dual practice in public and private sectors
Improving work environments (five dimensions of recurrent change: supervisor, task, colleagues, working hours, or location)	Job satisfaction increased, less burnout, less sickness-related absence. Changes perceived both as positive and as negative by employees, with some preferring change and others preferring stability
Financial and incentive mechanisms, such as providing financial rewards for good attendance	Sometimes effective, others inconclusive
Health intervention mechanisms, such as vaccination and exercise programs that aim at reducing work-related ill-health and absence	Was effective in reducing absenteeism where the program was prolonged and by including vaccination for seasonal prolonged epidemics
Mandatory attendance and surveillance of absenteeism behavior during disaster	Ethical concerns against public liberties

## Discussion

In relation to our first question on the pattern of interventions, from this overview, it is clear that a wide range of interventions has been tested in high- and middle-income countries to address the challenges of training, recruiting, retaining, and optimizing the performance from their health workforce. Some topics are more deeply documented than others—there is, for example, a large number of studies on HRH planning, educational interventions, and policies to reduce in-migration, but much less on topics such as HRH financing and task shifting.

On the strength of evidence supporting them (our second research question), it is clear that the evidence base supporting them varies—relatively few have been rigorously evaluated, which is surprising but also corroborated by other authors [4, 32, 53, 79, 134, 139]. Much of the literature is descriptive, rather than evaluative, reflecting the organic way in which many HRH reforms are introduced (and also, often, their contested nature). The wide range of contexts and challenges also makes it important to interpret the results carefully for new contexts. A more rigorous approach to testing HRH interventions would put this area of health systems strengthening on a par with others, where more rigorous evaluations are conducted (for example, on reforms to service delivery or financing modalities) [139].

In relation to different patterns across occupational groups, although some of the interventions have been more used and documented in relation to specific cadres, such as physicians or nurses, many of the lessons emerging from this evidence summary appear to apply across them, with many studies also highlighting the importance of tailoring to reflect individuals’ characteristics, such as age, location, and preferences.

Reflecting on the framework, we adopted a structure which starts from specific problems for HRH relating to production, recruitment and retention, distribution, and performance, but it is evident that some policy actions may address more than one area of challenge (for example, decentralized HR management may improve retention alongside performance), but equally that some policy actions may have conflicting results for different challenges (for example, supporting targeted in-migration to address shortages in some areas may reduce incentives to train domestic staff). Our categories overlap substantially with previous reviews [4] albeit with some difference (for example, we did not identify studies focusing on community engagement in HRH recruitment and planning, which may reflect market differences between LMICs and MHICs).

## Conclusion

This evidence review has examined published and grey evidence on interventions to attract, recruit, distribute, and manage an effective health workforce, focusing on physicians, nurses, and AHPs in high- and middle-income countries. Although attention is drawn to the relative lack of robust studies in some areas, there is sufficient accumulated descriptive and operational experience to indicate promising starting points to build an effective, equitable, and sustainable health workforce. It can also inform planners in low- and lower middle-income countries, who face many similar challenges but where the literature has to date focused on some topics (such as mixed retention packages and management of dual practice) [12] but less on others, such as attracting students to underserved specialties, deferring retirement, managing migration, and improving the effectiveness of CPD.

## Data Availability

The datasets used and/or analyzed during the current study are available from the corresponding author on reasonable request.

## Abbreviations

APN: Advanced practice nurse
CME: Continuous medical education
CPD: Continued professional development
EU: European Union
FWE: Full-time workload equivalent
GP: General practitioner
HiT: Health in transition
HR: Human resources
HRH: Human resources for health
HRIS: Human resource information systems
HRM: Human resource management
HWA: Health Workforce Australia
ICN: International Council of Nurses
IMG: International medical graduate
LTC: Long-term care
MPH: Masters in Public Health
NOSM: Northern Ontario School of Medicine
NP: Nurse practitioner
OECD: Organisation for Economic Co-operation and Development
P4P: Pay for performance
PIP: Practice Incentives Program
PSW: Personal support workers
RCS: Rural Clinical School
RN: Registered nurse
RPN: Registered practical nurse
UAE: United Arab Emirates
WISN: Workload Indicators of Staffing Needs

## Footnotes

1 These included midwives, pharmacists, general health workers, physiotherapists, dentists, occupational therapists, psychologists, dietitians, podiatrists, and public health workers; however, these terms and synonyms for HRH were not used during searching, which will have resulted in some missed studies.

2 Here too, additional terms could have been added, such as demand and supply. These would have potentially produced more hits, but given the broad scope of the review, our focus was on capturing the broad landscape, rather than aiming for comprehensiveness.
